# Prevalence and predictors of early blood transfusion in renal transplant recipients: a retrospective study

**DOI:** 10.1016/j.htct.2026.108547

**Published:** 2026-10-16

**Authors:** Pukar Chandra Shrestha, Kalpana Kumari Shrestha, Arati Rai, Dipesh Shrestha, Sumit Acharya, Rupesh Kumar Jha, Hari Prasad Baral, Rojan Adhikari, Bikash Khadka, Bikkin Dangol, Milan Shrestha, Lochan Shrestha, Sanisha Karki, Chahana Bogati, Puja Khadka, Binita Kutu, Sat Prasad Nepal

**Affiliations:** a Department of Transplant Surgery and Urology, Shahid Dharmabhakta National Transplant Center (SDNTC), Bhaktapur, Nepal; b Department of Nephrology, Shahid Dharmabhakta National Transplant Center (SDNTC), Bhaktapur, Nepal; c Department of Anesthesia, Shahid Dharmabhakta National Transplant Center (SDNTC), Bhaktapur, Nepal; d Department of Nursing, Shahid Dharmabhakta National Transplant Center (SDNTC), Bhaktapur, Nepal

**Keywords:** Blood transfusion, Blood products, Renal transplantation, Predictors, Resource limited settings

## Abstract

**Background:**

Anemia associated with chronic kidney disease is driven by reduced erythropoietin synthesis, chronic inflammation, and decreased red blood cell lifespan. During and after renal transplant surgery, recipients may need blood transfusions. Transfusion rates exhibit significant inter-center variability, largely driven by differing clinical criteria among transplant surgery, nephrology, and intensive care units.

**Study design and methods:**

A retrospective study was conducted of 407 renal transplant recipients who underwent transplantation from May 15, 2023 to August 16, 2025 at the Shahid Dharmabhakta National Transplant Center, Nepal. Early transfusion was defined as any blood product administration occurring within 14 days post-surgery. Clinical and Laboratory variables were compared between transfused and non-transfused patients using either the Mann–Whitney U test or chi-square test as appropriate. Variables significant in univariate analysis were included in multivariate logistic regression to identify predictors of transfusion.

**Results:**

Ninety-five patients (23.34%) required transfusions with leukoreduced red blood cells being the most common blood product. Univariate analysis revealed that preoperative hemoglobin levels, diabetic nephropathy, and the length of hospital stay were significantly associated with transfusion requirements. Multivariate analysis showed that preoperative hemoglobin and length of stay were predictors of transfusion. The hemoglobin nadir for the transfused group reached a mean of 6.3 ± 1.05 g/dL and a median of 6.1 g/dL, with an interquartile range from 4.2 to 10.0 g/dL.

**Discussion:**

The results of this study show that a conservative transfusion approach can be effective in achieving good clinical outcomes, especially in resource limited centers.

## Introduction

Anemia is a common problem in patients with chronic kidney disease (CKD). It is primarily caused by a reduction in the production of erythropoietin, a hormone that stimulates the formation of red blood cells. Other contributing factors include chronic inflammation, platelet dysfunction due to uremia, ongoing blood loss, and poor absorption of iron, folate, and vitamin B12 [[1], [2], [3], [4]]. These mechanisms often result in significant anemia prior to transplantation.

The use of erythropoiesis-stimulating agents (ESAs) has helped reduce the need for blood transfusions in CKD and transplant patients [3,5]. The Kidney Disease Outcomes Quality Initiative (KDOQI) and the Kidney Disease Improving Global Outcomes (KDIGO) guidelines recommend a hemoglobin (Hb) target of 10-11.5 g/dL for CKD patients receiving ESAs [5]. Thus, most CKD patients are anemic at the time of renal transplantation. However, anemia further worsens after renal transplantation due to surgical blood loss and the effects of immunosuppressive drugs [[2], [3], [4],^6^,^7^]. In these cases, transfusion may be necessary to manage symptomatic anemia and maintain adequate Hb levels.

Recent evidence suggests that early post-transplant transfusions may negatively impact graft outcomes. Several studies have reported associations with increased risk of donor-specific antibody formation, graft rejection, and even transplant failure [8,9]. Transfusion-related immunosuppression may also increase the risk of infections [10,11].

Although several studies have evaluated transfusion burden and predictors in renal transplantation, data from resource-limited transplant settings remain limited. To address this knowledge gap, the present study aimed to evaluate the transfusion burden within a single center, characterize the types of blood products utilized, and identify center-specific predictors of blood transfusion in a resource-limited transplant setting.

## Materials and methods

This is a retrospective cross-sectional study based on patient data from the renal transplant registry of a single institution. The Shahid Dharmabhakta National Transplant Center (SDNTC) is a high-volume National Transplant center for renal transplantation in Nepal. After obtaining ethical approval from the Nepal Health Research Council (NHRC - approval number 582-2025), records from the institutional archives were retrieved and analyzed using SPSS version 24.0 (IBM Corp., Armonk, NY, USA). Patients who received transfusions intraoperatively or within 14 days post-transplantation were identified and compared with patients who did not receive transfusions. The 14-day threshold for 'early' transfusion was adopted based on a large cohort study by Gaiffe et al., which reported that 28% (3,483/12,559) of patients received transfusions within two weeks of transplant surgery. The authors reported that early post-transplant transfusion was associated with increased risk of transplant failure [8].

The following variables were collected: recipient demographics (age, gender, and comorbidities); immunological data, including panel reactive antibody (PRA) I and II, human leukocyte antigen (HLA) mismatches, and immunosuppressant regimens; and surgical factors such as the presence of single versus multiple arteries, total warm ischemia time, and total cold ischemia time. Perioperative data were also recorded, including preoperative laboratory values (Hb, WBC count, platelets, urea, and INR), intraoperative and postoperative transfusion requirements, and the use of postoperative anticoagulation (LMWH or unfractionated heparin). Additionally, the total length of hospital stay was documented.

Continuous variables were assessed for normality using the Shapiro-Wilk test. Normally distributed variables were presented as mean ± standard deviation (SD), while non-normally distributed variables were expressed as median with interquartile range (IQR). For univariate analysis, continuous variables were compared using either the t-test or the Mann–Whitney test, depending on the normality of the data, while categorical variables were compared using the chi-square test.

A p-value <0.05 was considered statistically significant. Variables that were statistically significant in the univariate analysis were subsequently included in the multivariate logistic regression model to identify independent predictors of blood transfusion. Results were reported as odds ratios (OR) with 95% confidence intervals (95% CI).

Hb levels were recorded at multiple time points through patient admission and post-surgery till discharge. For transfused patients, the Hb nadir refers to the lowest Hb value at which the blood transfusion was administered during or after surgery. In non-transfused patients, the Hb nadir represented the lowest Hb value recorded during hospitalization after the transplant surgery till discharge.

## Results

Of the 407 kidney transplant recipients (405 live donors and 2 deceased donors), 95 patients (23.34%) required transfusion. Among the total recipients, 77 (18.9%) were female, and 330 (81.1%) were male.

These transplant recipients were relatively young with a median age of 37 years (range: 14-69) in the transfused group and 36 years (range: 17-66) in the non-transfused group. The majority of patients (n = 340; 83.5%) were between 21 and 50 years of age. Two deaths were observed of the 407 patients: one patient died due to dengue hemorrhagic fever, and another due to septic shock.

Postoperatively, enoxaparin was administered to patients with a single arterial anastomosis, whereas unfractionated heparin was used for those with multiple arterial anastomoses. Among the 364 patients who received LMWH, 83 (22.8%) received blood transfusions, whereas 12 (27.9%) of the 43 patients who received unfractionated heparin required transfusions.

Leukoreduced red blood cells (LR-RBC) were transfused in 91 patients (22.35%), red blood cells (RBC) in 17 patients (4.18%), fresh frozen plasma (FFP) in eight patients (1.96%), platelet-rich plasma (PRP) in three patients (0.7%), and cryoprecipitate in one patient (0.2%). Of the 230 units transfused during the study period, 163 (70.9%) were LR-RBC, 33 (14.3%) RBC, 20 (8.7%) FFP, five (2.2%) PRP, and nine (3.9%) cryoprecipitate (Table 1).

**Table 1:** Utilization of blood components among renal transplant recipients (n = 407).

Blood Product	Patients transfused n (%)	Units Transfused n (%)
LR-RBC	91 (22.35)	163 (70.87)
RBC	17 (4.18)	33 (14.35)
FFP	8 (1.96)	20 (8.7)
PRP	3 (0.7)	5 (2.2)
Cryoprecipitate	1 (0.2)	9 (3.9)
		-
Overall Utilization	95 (23.34)	230

LR-RBC: Leukoreduced red blood cells; RBC: Red blood cells; FFP: Fresh frozen plasma; PRP: Platelet rich plasma

Hypertensive nephropathy was the most common cause of CKD in the institution, accounting for 314 patients (77.2%), followed by IgA nephropathy (n = 51; 12.5%) and diabetic nephropathy (n = 31; 7.6%) (Table 2).

**Table 2:** Baseline causes of end-stage renal disease in renal transplant recipients (n = 407).

Causes of CKD	n (%)
Hypertensive nephropathy	314 (77.2)
IgA nephropathy	51 (12.5)
Diabetic nephropathy	31 (7.6)
FSGS	8 (2.0)
SLE nephropathy	2 (0.5)
Unknown	1 (0.2)

CKD: Chronic kidney disease; FSGS: Focal Segmental Glomerulosclerosis; SLE: Systemic Lupus Erythematosus

Acute tubular necrosis was identified in 13 cases (3.2%). Additionally, there were six cases of histopathological rejection, consisting of three cases (0.7%) of antibody mediated rejection and three cases of acute cellular rejection.

Univariate analysis showed that preoperative hemoglobin, diabetic nephropathy, and length of stay were significantly associated with the need for transfusion (Table 3). Moreover, preoperative Hb, baseline platelet count, diabetic nephropathy, warm ischemia time, and length of stay were identified as significant factors for patients receiving LR-RBC transfusions (Table 4).

**Table 3:** Univariate analysis comparing patients with and without blood transfusion.

Variable	Transfused	Non-transfused	p-value
Demographic variables			
n (%)	95 (23.34 %)	312 (76.7%)	
Age* - median (IQR)	37 (49)	36 (55)	0.146
Gender - n			
Male	73	257	
Female	22	55	0.228
Comorbidities - n			
HTN	70	244	0.358
DM	13	18	0.011
IgA N	11	40	0.749
SLE	0	2	0.437
FSGS	0	8	0.115
Preoperative laboratory variables - median (IQR)			
Preoperative Hb*	9.7 (8.00)	10.40 (9.30)	0.009
Preoperative TLC*	5650 (12200)	5860 (13670)	0.171
Preoperative Platelets*	158500 (624680)	183000 (431922.10)	0.050
Preoperative Urea*	103.0 (159.8)	103.8 (238.9)	0.599
Preoperative INR* Missing - 4	0.8 (0.5)	0.8 (11.3)	0.559
Immunologic factors			
HLA mismatches - n			
0	3	18	
1	3	21	
2	21	46	
3	31	80	
4	10	48	
5	13	49	
6	4	21	
Missing - 39			0.207
PRA I - n			
0%	55	190	
1%-50%	33	104	
≥51%	5	7	
Missing - 13			0.306
PRA II - n			
0%	48	175	
1%-50%	40	117	
≥51%	5	11	
Missing - 11			0.506
Transplant-related factors			
Immunosuppressant - n			
Simulect	14	62	
Grafalon	51	132	
ATG	30	117	
Missing - 1			0.148
Arteries - n			
Single artery	84	280	
Multiple arteries	11	32	0.713
Total WIT * - median (IQR) Missing - 8	30 (50)	29 (220)	0.072
CIT* - median (IQR) Missing - 4	63 (167)	61 (286)	0.102
Postoperative variables			
Anticoagulants after surgery - n			
Enoxaparin	83	281	
Heparin	12	31	0.454
Post-op days* - median (IQR)	7 (21)	5 (22)	<0.001

Continuous variables are expressed as median (Interquartile range - IQR) and compared using the Mann–Whitney U test*. Categorical variables were compared using the chi-square test.

HTN: Hypertension; DM: Diabetes mellitus; IgA N: IgA nephropathy; Hb: Hemoglobin; TLC: Total leukocyte count; INR: International normalized ratio; HLA: Human leukocyte antigen; PRA: Panel reactive antibody; IS: Immunosuppressants; ATG: Anti-thymocyte Globulin; WIT: Warm ischemia time; CIT: Cold ischemia time

**Table 4:** Univariate analysis comparing patients with and without leukoreduced red blood cell (LR-RBC) transfusion.

Variable	LR-RBC	No LR-RBC	p-value
Total patients – n (%)	91(22.35)	316 (77.6)	
Demographic variables			
Age* – median (IQR)	36 (49)	36 (55)	0.234
Gender – n (%)			
Male	72	258	
Female	19	58	0.588
Comorbidities – n (%)			
HTN	66	248	0.233
DM	13	18	0.006
IgA N	11	40	0.885
SLE	0	2	0.450
FSGS	0	8	0.125
Preoperative variables – median (IQR)			
Preoperative Hb*	9.7 (8)	10.4 (9.3)	0.001
Preoperative TLC*	5650 (12200)	5860 (13670)	0.223
Preoperative Platelets*	161500 (624680)	182000 (431922)	0.037
Preoperative Urea*	103.0 (159.8)	103.8 (238.9)	0.592
Preoperative INR* Missing - 4	0.8 (0.5)	0.8 (11.3)	0.798
Immunologic variables			
HLA mismatches - n			
0			
1	3	18	
2	3	21	
3	20	47	
4	30	81	
5	10	48	
6	12	50	
Missing - 39	4	21	0.278
PRA I - n			
0%	54	191	
1%-50%	31	106	

≥51%	5	7	
Missing - 13			0.286
PRA II - n			
0%	47	176	
1%-50%	38	119	
≥51%	5	11	
Missing - 11			0.548
Transplant variables			
Immunosuppressant - n			
Simulect	13	63	
Grafalon	49	134	
ATG	29	118	
Missing - 1			0.146
Arteries - n			
Single artery	80	284	
Multiple arteries	11	32	0.592
Total WIT * – median (IQR) Missing - 8	30 (50)	29 (220)	0.046
CIT* – median (IQR) Missing - 4	62 (167)	61 (286)	0.358
Postoperative variables			
Anticoagulants after surgery - n			
Enoxaparin	79	285	
Heparin	12	31	0.356
Post-op days* – median (IQR)	6.5 (21)	5 (22)	<0.001

Continuous variables are expressed as median (Interquartile range - IQR) and compared using the Mann–Whitney U test*. Categorical variables were compared using the chi-square test.

LR-RBC: Leukoreduced red blood cells; HTN: Hypertension; DM: Diabetes mellitus; IgA N: IgA nephropathy; Hb: Hemoglobin; TLC: Total leukocyte count; INR: International normalized ratio; HLA: Human leukocyte antigen; PRA: Panel reactive antibody; IS: Immunosuppressants; ATG: Anti-thymocyte globulin; WIT: Warm ischemia time; CIT: Cold ischemia time

Multivariate analysis identified lower preoperative Hb and longer length of stay as independent predictors of transfusion in these renal transplant patients. Specifically, each 1 g/dL increase in preoperative Hb was associated with a 23.2% reduction in the odds of blood transfusion (Table 5).

**Table 5:** Multivariate logistic regression analysis for predictors of blood transfusion and leukoreduced red blood cell (LR-RBC) transfusion.

	Blood Transfusion			LR-RBC Transfusion		
Variable	OR	95% CI	p-value	OR	95% CI	p-value
Preoperative Hb	0.768	0.652–0.904	0.002*	0.766	0.648–0.907	0.002⁎
DM	0.537	0.232–1.156	0.128	0.543	0.241–1.268	0.161
WIT total	-	-	-	1.000	0.985–1.015	0.949
Preoperative Platelets	-	-	-	1.000	1.000–1.000	0.193
Length of stay (Days)	1.155	1.081–1.235	<0.001⁎	1.150	1.075–1.230	<0.001⁎

OR: Odds ratio; 95% CI: 95% Confidence interval; LR-RBC: Leukoreduced red blood cell; Hb: Hemoglobin; DM: Diabetes mellitus; WIT: Warm ischemia time

⁎ Statistically significant (p-value <0.05)

By multivariate analysis for LR-RBC transfusions, preoperative Hb and postoperative length of stay were statistically significant (Table 5). Univariate and multivariate analyses were not performed for the other blood products due to the small sample size.

Examining the Hb level at which blood transfusions was performed, the mean nadir in the transfused group was 6.3 ± 1.05 g/dL and the median nadir was 6.1 g/dL (IQR: 4.2–10.0). In non-transfused patients, the mean Hb nadir was 8.1 ± 1.46 g/dL, and the median nadir was 7.9 g/dL (IQR: 5.2–12.9).

Among the renal transplant recipients, 46 of 65 patients (70.8%) with a nadir Hb ≤6 g/dL required transfusions. When the Hb nadir was between 6.1 and 7 g/dL, 31 of 86 patients (36%) were transfused. In contrast, only 18 of 255 patients (7.1%) with a Hb nadir >7 g/dL received transfusions. These findings reflect the conservative transfusion approach maintained at this institution

## Discussion

Blood transfusions in the perioperative period of major surgeries have been associated with increased morbidity and mortality [6]. Transfusion-related complications include infections, circulatory overload, and transfusion-related lung injury [12]. Mazzeffi et al. found that transfused renal transplant recipients had a ninefold higher risk of developing sepsis compared to non-transfused patients [13]. Red blood cell transfusions may also suppress lymphocyte function, possibly due to immunomodulatory effects of donor leukocytes [10,11]. In renal transplant recipients, anemia is common and often necessitating transfusion due to both the underlying effects of chronic kidney disease and intraoperative blood loss [14]. Given the potential risks associated with transfusion, understanding transfusion practices and rates across transplant centers is clinically important.

Differences in transfusion rates across centers may reflect variations in transfusion protocols, whether restrictive or liberal [12]. In a large French study involving 12,559 renal transplant patients, 3,483 (28%) received blood transfusions [8]. Another study reported transfusions in 468 out of 1,258 recipients (37.2%), with a higher risk of infection observed in transfused patients during a median follow-up of 3.8 years [11]. In a study by Kakkar et al. of 510 transplant recipients, 47.3% (n = 241) received transfusions [12] Similarly, Daloul et al. reported that 47% (127/273) of renal transplant recipients received transfusions [15].

This study center adheres to a conservative transfusion protocol, wherein clinicians exercise clinical judgment to initiate transfusion only in response to changes in vital signs or significant blood loss leading to hemodynamic instability, rather than relying solely on restrictive Hb thresholds. No graft failures were observed prior to hospital discharge, and the mortality rate was minimal. This approach to transfusion may help minimize unnecessary procedures while maintaining favorable short-term outcomes.

In the present study, 99.5% of the recipients received kidneys from living donors. As most donors were living, the risk of perioperative bleeding may be lower compared to deceased donor transplantations. In a study by Hachem et al., 59 out of 1203 (4.9%) kidney transplant patients had post-transplant surgical site hemorrhages with most of these cases (89.8%) occurring within one day post-surgery. The deceased donor group contributed to 72.9% of the recipients with hemorrhages.

The authors attributed this to several factors. Living donor kidneys undergo perinephric hemostasis during graft mobilization. In contrast, deceased donor kidneys are removed en bloc with surrounding tissues, and hemostasis is achieved only after reperfusion. Deceased donor kidneys may also have fragile tissues due to comorbidities, older age, and higher body mass index. In addition, supratherapeutic anticoagulation is sometimes given to deceased donors before organ retrieval. Furthermore, recipients of deceased donor kidneys often have longer waiting list duration and prolonged dialysis exposure, which may affect tissue and vessel integrity [16].

At this institution, 23.34% of patients (95/407) received transfusions, a rate comparatively lower than those reported in the literature. For instance, while these findings were similar to the 26.10% reported by Marzouk et al. [17], they stand in contrast to the significantly higher rates observed by Rodriguez et al. [5] (39.00%) and Makroo et al. [12] (47.30%). Similarly, Gaiffe et al. [8] reported a rate of 28.00% in a cohort of over 12,000 patients, and Azarniouch et al. [11] observed a rate of 37.20% in 1,258 patients. This discrepancy might be attributed to our conservative transfusion strategy, the relative young patient population, the predominance of live donors, and the limited sample size.

A unique feature in the current study was the level of the Hb nadir at which the patients were transfused. The mean Hb nadir at the time of transfusion, regardless of the postoperative day, was 6.3 g/dL, and the median Hb was 6.1 g/dL. Markouz et al. reported a mean Hb of 6.9 g/dL in recipients of deceased donor kidneys [17]. In another study, which included both living and brain-dead donors, the median Hb before RBC transfusions was 7.8 g/dL [18]. Jouve et al. started transfusions when the Hb fell below 8 g/dL in non-heart disease patients and 10 g/dL for heart disease patients; the median Hb level for transfusion was 8.3g/dL [19]. The mean Hb at the time of transfusion reported by Bukhari et al. was 7.4 g/dL [20], while Daloul et al. reported a median Hb of 7.1 g/dL at transfusion [15].

In comparison with these studies, the Hb level at which transfusions were initiated in the present cohort was lower. This finding is consistent with the conservative transfusion strategy followed at the center, which may reduce unnecessary transfusions while maintaining favorable clinical outcomes through to hospital discharge.

The lower Hb threshold for transfusion observed in this study may be explained by the relatively younger patient population, which is generally more tolerant to anemia. The median age of patients undergoing transfusions at the institution was 37 years old. Therefore, the transfusion could be safely withheld until Hb levels were as low as 7 g/dL, provided that vital signs remained stable and patients were hemodynamically stable. Adequate oxygen delivery depends on cardiac output, Hb concentration, and oxygen saturation. Because cardiac output declines with aging, older patients are more vulnerable to hypoxia during anemic states. In older patients due to cardiovascular problems, medications like beta blockers and calcium channel blockers further compromise the increase in cardiac output required to maintain oxygen diffusion to tissues when the Hb level drops [21]. Simon et al. demonstrated comparable oxygen-carrying capacity in a younger cohort (<40 years) with an Hb of 7 g/dL and an elderly population (>60 years) with an Hb of 10 g/dL [21]. Thus, the authors suggested that Hb thresholds should be different for transfusions in the old versus young populations.

Furthermore, to enhance hemostasis intraoperatively and at the end of surgery, this institution routinely uses topical hemostatic agents such as oxidized regenerated cellulose (e.g., surgicel) and fibrin sealants. In some patients, the use of ESAs are encouraged; this may also have helped prevent the need for transfusions.

The American Society of Anesthesiologists (ASA) Task Force recommends initiating transfusions at Hb thresholds between 6 g/dL and 10 g/dL based on factors such as ongoing bleeding, vital signs, intravascular volume status, evidence of organ ischemia, cardiopulmonary reserve, ST-segment changes on ECG, urine output, and arterial blood gas findings [22]. The Association for the Advancement of Blood and Biotherapies (AABB) recommends a restrictive RBC transfusion threshold of Hb 7 g/dL for hemodynamically stable hospitalized adult patients, along with critically ill patients. This approach leads to a decrease in the number of transfusions and expense, and avoids associated adverse effects [23]. No such guidelines exist in the case of renal transplantation [14] and the long-term effects of transfusion on graft survival must be taken into consideration [8,9,14].

The concept of a restrictive transfusion approach aligns with that of patient blood management (PBM). PBM emphasizes a patient's clinical needs, minimizing unnecessary transfusions, optimizing the patient's own blood volume and function, and employing alternatives (like iron therapy, erythropoiesis-stimulating agents, and surgical techniques) to reduce reliance on donor blood [24,25]. Leahy et al. found that implementing the PBM concept led to a decrease in blood product utilization and a reduced length of stay. Furthermore, mean pre-transfusion Hb levels declined from 7.9 g/dL to 7.3 g/dL by the sixth year of the study across various departments in four tertiary hospitals [24].

In Nepal, most hospitals, including our transplant center, do not maintain separate blood banks. Patients often need to obtain blood from other hospitals, the Red Cross, or laboratory blood banks. For leukoreduced blood products, patient families must also arrange donors and procure leukocyte-depleting filters, adding logistical challenges. The National Red Cross Society (NRCS) provides approximately 90% of transfusion services [26]. However, the increasing numbers of hospitals, medical colleges, and nursing homes have led to a growing demand for blood products. Whole blood donation rates in high-income countries average 32.6 per 1,000 population, compared to 6.9 per 1,000 in low-income countries, and only 4.4 per 1,000 in Nepal [26]. These limitations directly impact transfusion practices and necessitate careful planning in transplant settings.

Solid organ transplant surgeries are complex and often involve significant blood loss. To ensure safe surgical planning and optimal patient care, it is important to assess transfusion requirements and identify factors that contribute to increased blood product use [12]. In this study, preoperative Hb levels and postoperative length of stay were significant predictors of transfusion (Table 5), highlighting the importance of the preoperative assessment and targeted perioperative management. Understanding these predictors can help clinicians anticipate transfusion needs, reduce unnecessary blood use, and improve patient outcomes in resource-limited settings like Nepal.

Previous studies have identified several predictors of blood transfusion in renal transplant recipients. In one study, hypertensive donors and recipient factors such as older age, female sex, non-white ethnicity, and dialysis dependence were associated with transfusion [14]. Similarly, Marzouk et al. identified several predictors of transfusion in recipients of deceased donor kidneys, including recipient age >55 years, male sex, and Hb <11.5 g/dL [17]. Kang et al. also found that preoperative Hb level, female recipient, and anti-thymocyte globulin induction were associated with early postoperative transfusions [27]. Daloul et al. reported that older recipients, deceased donors, and basiliximab induction were associated with transfusions [15].

In transplants involving deceased donors, anticoagulant use and male donor sex were associated with higher transfusion rates, whereas in living donor transplants anticoagulants did not appear to influence transfusion need [17]. At our center, anticoagulation is given after surgery using either enoxaparin or intravenous heparin. In the present study, anticoagulant use was not associated with transfusion risk (Table 3).

Regarding hospital stay, transplant patients at our institution are usually discharged on the fifth postoperative day. Some patients remained hospitalized longer than scheduled due to complications or because they could not immediately arrange accommodation, as many patients live outside the city. The most common complications were high drain output, urinary tract infection, and acute tubular necrosis (Supplementary Table S1).

Although length of hospital stay showed statistical significance in predicting transfusion, most transfusions (97.9%) occurred within the first three postoperative days. Therefore, the association between hospital stay and transfusion in this study may not be clinically significant.

The main limitation of the current study is the absence of data on long-term graft-related outcomes of recipients who received transfusions. Previous studies have suggested possible associations between transfusions and graft-related outcomes. However, the lack of a long-term follow-up in this study limits our ability to determine whether the observed transfusion patterns influenced long-term graft prognosis.

This is a retrospective study; the sample size was limited although all the eligible kidney transplant recipients during the study period were included. This study is based on a single-center experience; transfusion protocols, surgical techniques, and postoperative care vary across institutions and countries.

Our center follows a conservative transfusion approach, which may differ from more liberal practices in other institutions and may therefore influence transfusion rates. Therefore, our findings may not be generalizable to transplant centers with different protocols and resources. In addition, transfusion decisions are based on the clinician’s judgement.

Finally, several relevant variables were not studied. Information regarding estimated blood loss, preoperative anticoagulant use, and pre-transplant dialysis history was not available for this analysis. Despite these limitations, this study provides valuable real-world data on transfusion practices in a resource-limited transplant setting, highlighting a restrictive approach and identifying key predictors that may assist clinical decision-making

## Conclusion

This study provides real-world data on transfusion practices in a resource-limited transplant setting. A lower transfusion rate (23.34%) and Hb nadir threshold were observed compared with published reports, reflecting the center’s conservative transfusion policy, younger recipient population, and predominance of living donor transplants. Preoperative Hb and postoperative length of stay were significant predictors of transfusion, underscoring the importance of a careful preoperative assessment and perioperative management. Although these associations should be interpreted with caution given the retrospective design, the findings identify key factors that may guide restrictive transfusion strategies and patient blood management in similar clinical settings.

## Funding sources

This research did not receive any specific grant from funding agencies in the public, commercial, or not-for-profit sectors.

## Conflicts of interest

The author declares no conflicts of interest.

## Data Availability

The data supporting this study findings are available from the corresponding author upon reasonable request. The data is not publicly available due to ethical and institutional restrictions.

## Appendix Supplementary materials

Image, application 1

## References

[1] Fishbane S., Spinowitz B. (2018). Update on anemia in ESRD and earlier stages of CKD: core curriculum 2018. Am J Kidney Dis.

[2] Burton H., Iyamu-Perisanidou L., Steenkamp R., Evans R., Mumford L., Evans K.M (2019). Causes of renal allograft failure in the UK: trends in UK renal registry and national health service blood and transplant data from 2000 to 2013. Nephrol Dial Transplant.

[3] Leffell M.S, Kim D., Vega R.M, Zachary A.A, Petersen J., Hart J.M (2014). Red blood cell transfusions and the risk of allosensitization in patients awaiting primary kidney transplantation. Transplantation.

[4] Vanrenterghem Y., Ponticelli C., Morales J.M, Abramowicz D., Baboolal K., Eklund B. (2003). Prevalence and management of anemia in renal transplant recipients: a European survey. Am J Transplant.

[5] Rodríguez-Espinosa D., Broseta J.J, Rosario A., Cacho J., Tena B., Cuadrado-Payán E. (2024). Challenges in perioperative blood transfusions in kidney transplantation and the need for patient blood management. Blood Transfus.

[6] Gonzalez Gutierrez F., Sun J., Sambandam S. (2023). Risk of blood transfusion following total hip arthroplasty: a national database study from 367,894 patients. J Orthop.

[7] Verghese P., Gillingham K., Matas A., Chinnakotla S., Chavers B. (2016). Post-transplant blood transfusions and pediatric renal allograft outcomes. Pediatr Transplant.

[8] Gaiffe E., Vernerey D., Bardiaux L., Leroux F., Meurisse A., Bamoulid J. (2022). Early post-transplant red blood cell transfusion is associated with an increased risk of transplant failure: a nationwide French study. Front Immunol.

[9] Scornik J.C, Schold J.D, Bucci M., Meier-Kriesche H.U (2009). Effects of blood transfusions given after renal transplantation. Transplantation.

[10] Fischer E., Lenhard V., Seifert P., Kluge A., Johannsen R. (1980). Blood transfusion-induced suppression of cellular immunity in man. Hum Immunol.

[11] Massicotte-Azarniouch D., Sood M.M, Fergusson D.A, Chassé M., Tinmouth A., Knoll G.A. (2021). Blood transfusion and adverse graft-related events in kidney transplant patients. Kidney Int Rep.

[12] Makroo R.N, Kakkar B., Chowdhry M., Nayak S., Bhatia A. (2016). Retrospective analysis of perioperative transfusion requirements in living donor renal transplantation. Transfus Apher Sci.

[13] Mazzeffi M., Jonna S., Blanco N., Mavrothalassitis O., Odekwu O., Fontaine M. (2018). Intraoperative red blood cell transfusion, delayed graft function, and infection after kidney transplant: an observational cohort study. J Anesth.

[14] Hassan S., Gleeson S., Thomson T., Spensley K.J, Dor F., Brown C. (2023). Clinical impact of early post-transplant red cell transfusions in kidney transplantation: a systematic review and meta-analysis. Front Transplant.

[15] Daloul R., Braga J.R, Diez A., Logan A., Pesavento T. (2021). Early posttransplant blood transfusion and risk for worse graft outcomes. Kidney Int Rep.

[16] Hachem L.D, Ghanekar A., Selzner M., Famure O., Li Y., Kim S.J. (2017). Postoperative surgical-site hemorrhage after kidney transplantation: incidence, risk factors, and outcomes. Transpl Int.

[17] Marzouk K., Lawen J., Kiberd B.A. (2013). Blood transfusion in deceased donor kidney transplantation. Transplant Res.

[18] de Hilla N.OR., Etxarri N.M, Martín M.F, Abaunza de Juan O., Rodríguez Davis M.P, Vila I Noguer R. (2024). Characterization of patients needing red blood cell transfusion after kidney transplant. Nephrol Dial Transplant.

[19] Jouve T., Noble J., Naciri-Bennani H., Dard C., Masson D., Fiard G., Malvezzi P., Rostaing L. (2022). Early blood transfusion after kidney transplantation does not lead to dnDSA development: the BloodIm study. Front Immunol.

[20] Bukhari M.A, Alhomayani F.K, Al Eid H.S, Al-Malki N.K, Alotaibi M.E, Hussein M.A (2023). Is peri-transplant blood transfusion associated with worse transplant outcomes? A retrospective study. World J Transplant.

[21] Simon G.I, Craswell A., Thom O., Chew M.S, Anstey C.M, Fung Y.L. (2019). Impacts of aging on anemia tolerance, transfusion thresholds, and patient blood management. Transfus Med Rev.

[22] (2015). Practice guidelines for perioperative blood management. Anesthesiology.

[23] Carson J.L, Guyatt G., Heddle N.M, Grossman B.J, Cohn C.S, Fung M.K (2016). Clinical practice guidelines from the AABB: red blood cell transfusion thresholds and storage. JAMA.

[24] Leahy M.F, Hofmann A., Towler S., Trentino K.M, Burrows S.A, Swain S.G (2017). Improved outcomes and reduced costs associated with a health-system-wide patient blood management program: a retrospective observational study in four major adult tertiary-care hospitals. Transfusion.

[25] Isbister J.P. (2013). The three-pillar matrix of patient blood management – an overview. Best Pract Res Clin Anaesthesiol.

[26] Paudel P., Nepal B. (2025). Blood transfusion services in Nepal: an overview. Asian J Transfus Sci.

[27] Kang M., Koh H.H, Yim S.H, Choi M.C, Kim H.J, Kim H.W (2025). Clinical implications of early blood transfusion after kidney transplantation. Sci Rep.

