# Supplementary material for: Prevalence and predictors of early blood transfusion in renal transplant recipients: a retrospective study
Source: Hematol Transfus Cell Ther. 2026 Oct 16;48(4):108547. doi: 10.1016/j.htct.2026.108547 (PMC13694864; doi:10.1016/j.htct.2026.108547)
Supplement: Supplementary file 1 [file mmc1.docx]

**Supplementary Table S1:** Distribution of clinical and non-clinical reasons for prolonged hospital stay in patients receiving blood transfusions (n = 95).

| **Variable** | **Up to 5 POD** | **6 -10 POD** | **11 – 14 POD** | **≥ 15 POD** |
| --- | --- | --- | --- | --- |
| High Drain output |  | 8 |  | 1 |
| Fever and High TLC | 1 | 3 |  |  |
| Slow decrease in Cr |  | 4 | 1 | 1 |
| Low TLC |  | 1 | 1 |  |
| UTI |  | 6 | 2 |  |
| High RBS |  | 3 |  |  |
| High Urine output |  | 2 | 1 |  |
| DGF + ATN |  | 1 |  | 1 |
| ATN |  | 1 | 4 | 3 |
| High TLC |  | 5 |  |  |
| Increase Cr |  | 1 | 3 |  |
| Ureteroneocystostomy |  |  |  | 1 |
| Re-exploration |  | 6 |  | 1 |
| AMBR |  | 1 |  | 2 |
| ACR |  |  |  | 2 |
| Wound Infection |  | 1 | 1 |  |
| RVT |  |  | 1 |  |
| Abnormal Coagulation |  |  | 1 |  |
| Central line infection | 2 | 1 | 1 | 1 |
| Loose stools |  | 1 |  |  |
| Psychiatric condition |  | 1 |  |  |
| Pulmonary edema |  | 1 |  |  |
| Dengue Hemorrhagic fever |  | 1 |  |  |
| Urine leakage |  | 1 |  |  |
| HTN |  | 1 |  |  |
| Pneumonia |  | 2 |  |  |
| Partial small bowel obstruction |  | 1 |  |  |
| Scrotal swelling |  | 1 |  |  |
| Blood C/S positive | 1 |  |  |  |
| Clot evacuation | 1 |  |  |  |
| Acute pancreatitis |  | 2 |  |  |
| Delirium | 1 |  |  |  |
| Sepsis |  |  |  | 1 |
| Unable to find residence |  | 3 |  |  |
| Total: 95 | 6 | 59 | 16 | 14 |

POD: Postoperative day; TLC: Total leukocyte count; Cr- Creatinine; UTI: Urinary tract infection; RBS: Random blood sugar; DGF: Delayed graft function; ATN: Acute tubular necrosis; ABMR: Antibody mediated rejection; ACR: Acute cellular rejection; RVT: Renal vein thrombosis; HTN: Hypertension; C/S: Culture and sensitivity
